# Speckle tracking derived strain in neonates: planes, layers and drift

**DOI:** 10.1007/s10554-021-02200-8

**Published:** 2021-03-12

**Authors:** Umael Khan, Tom R. Omdal, Knut Matre, Gottfried Greve

**Affiliations:** 1 Department of Clinical Science, University of Bergen, Jonas Lies veg 87, 5021 Bergen, Hordaland Norway; 2 Department of Heart Disease, Haukeland University Hospital, Bergen, Norway

**Keywords:** Speckle tracking echocardiography, Global longitudinal strain, Multilayer, Layer specific strain, Drift compensation

## Abstract

The aims of this study was to assess the effect of using a four chamber versus a three plane model on speckle tracking derived global longitudinal strain, the effects of drift compensation, the effect of assessing strain in different layers and finally the interplay between these aspects for the assessment of strain in neonates. Speckle tracking derived longitudinal strain was obtained from 22 healthy neonates. ANOVA, Bland–Altman analyses, coefficients of variation and assessment of intraclass correlation coefficients were conducted to assess the effect of the abovementioned aspects as well as assess both inter-observer and intra-observer variability. Neither the use of the three plane model versus the four chamber model nor the use of drift compensation had a substantial effect on global longitudinal strain (less than 1%, depending on which layer was being assessed). A gradient was seen with increasing strain from the epicardial to endocardial layers, similar to what is seen in older subjects. Finally, drift compensation introduced more discrepancy in segmental strain values compared to global longitudinal strain. Global longitudinal strain in healthy neonates remains reasonably consistent regardless of whether the three plane or four chamber model is used and whether drift compensation is applied. Its value increases when one moves from the endocardial to the epicardial layer. Finally, drift compensation introduces more discrepancy for regional measures of longitudinal strain compared to global longitudinal strain.

## Introduction

STE derived strain measurements were introduced as a clinical measure in the early 2000s [1]. Since then, it has rapidly gained traction as a measure of left ventricular function, and is now included in adult echocardiographic guidelines [2]. In recent years, there has been increasing interest in application of strain measurements in the neonatal population since several studies indicate that strain is often a more sensitive measure of ventricular function than conventional echocardiography [3, 4]. Reference values in healthy neonates are currently being established, which will allow for the clinical assessment of pathological strain values [5, 6].

Optimal application of STE requires an understanding of which factors affect STE measurements. Lack of such understanding could lead to misinterpretation of strain values; changes in strain values due to image acquisition or processing factors could be falsely attributed to a change in cardiac function.

The most commonly assessed systolic strain parameter is global longitudinal strain (GLS). Most pediatric studies of GLS in healthy subjects only assess GLS in the four chamber (4ch) view [6–8]. However, clinical guidelines recommend that strain should be derived from all three apical views, namely four chamber (4ch), three chamber (3ch) and two chamber (2ch) views [2, 5]. The different assessments of GLS lead to the question of whether three plane GLS differs from 4ch GLS. This question is especially pertinent in neonates as reference values in healthy neonates are still being established. Furthermore, as neonates are frequently restless, it would be easier for a clinician to obtain 4ch GLS measurements than three plane GLS.

The ventricular wall can be divided into three software defined layers referred to as endocardial, midwall and epicardial layer. Studies assessing multilayer strain show a gradient with increasing strain values from the epicardial to the endocardial layer. The assessment of multilayer strain could be of clinical utility [9]. However, there is a shortage of studies that examine multilayer GLS in healthy neonates.

**Fig. 1:**
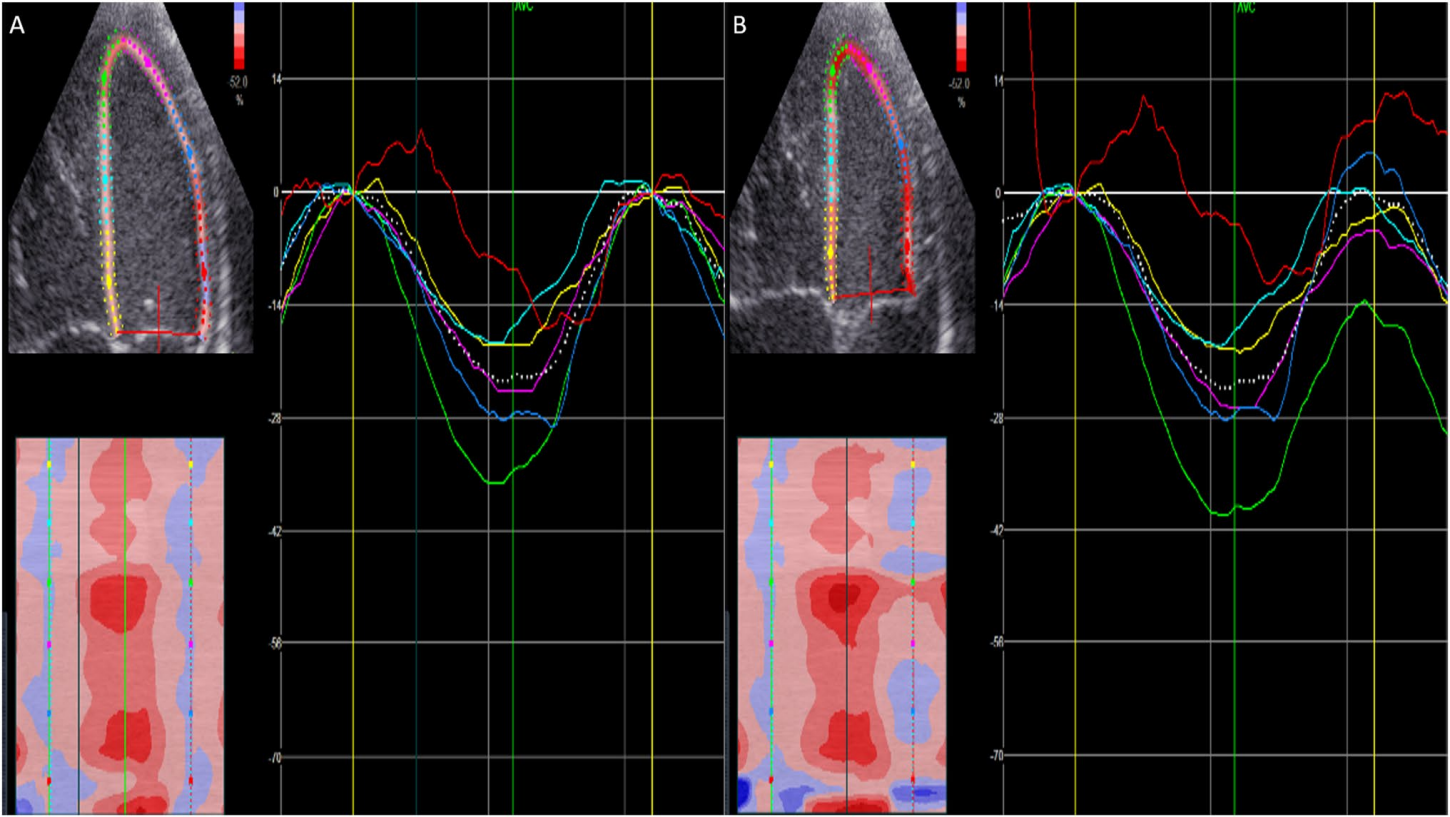
Speckle tracking echocardiography derived strain measurements from the four chamber view. Each of the curves represent strain in a specific segment, whereas the stapled line represents the average strain. The red circle shows how drift compensation results in strain curves returning back to baseline. **A** Drift compensation on (default); **B** drift compensation off

**Fig. 2:**
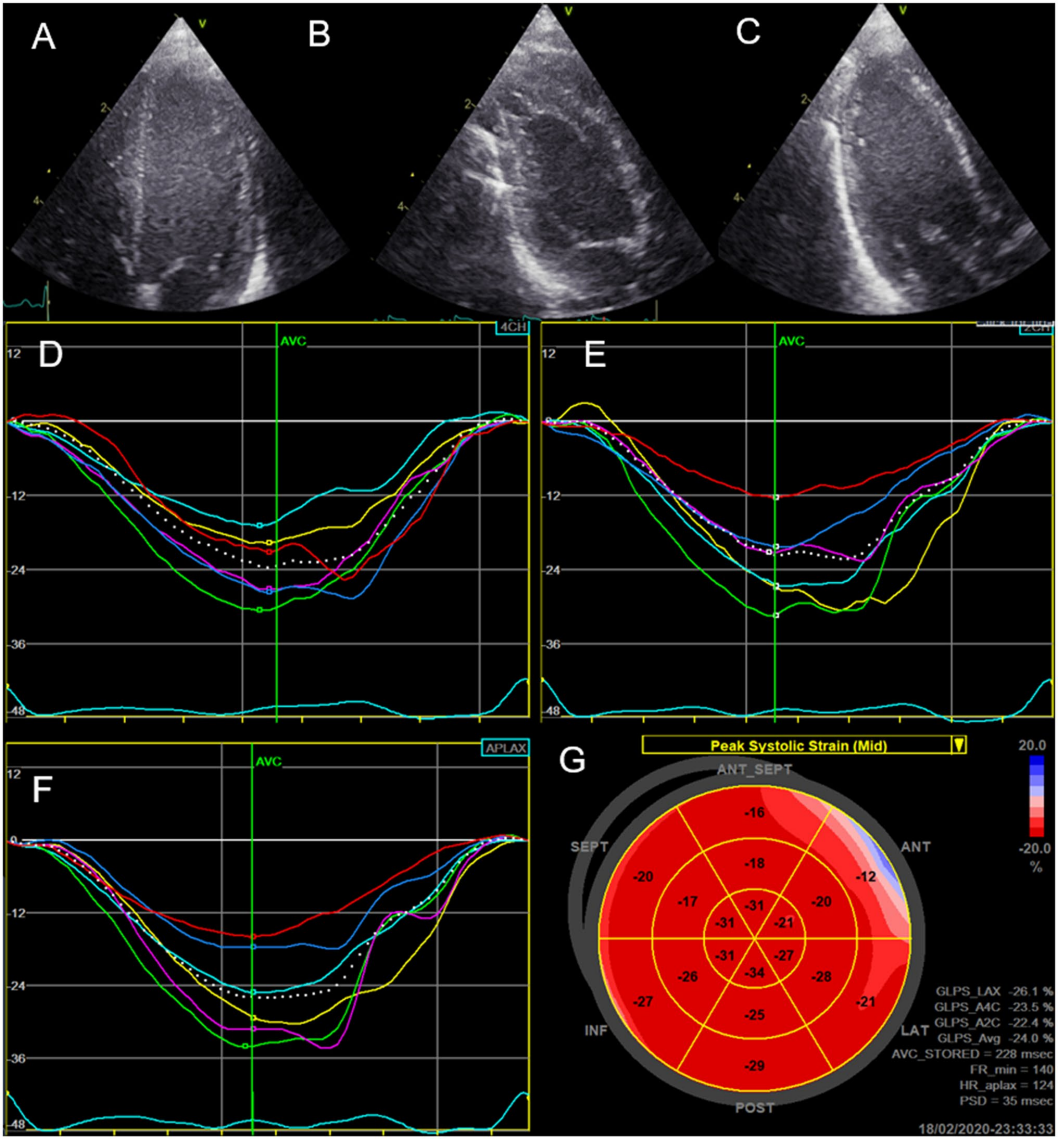
Speckle tracking echocardiography derived strain measurements from all three views, their respective strain curves and a combined bullseye plot showing segmental strain values in the midwall (corresponding graphs were also obtained for the endocardial and epicardial layers). **A** Four chamber view; **B** two chamber view; **C** three chamber view; **D** strain curves four chamber view; **E** Strain curves two chamber view; **F** strain curves three chamber view; **G** Bullseye plot presenting segmental strain values

In addition to imaging planes and layers, one must also take into account image processing. Due to the variability of STE strain measurements, previous studies have assessed the effect of image acquisition and processing parameters such as vendor heterogeneity, frame rate, frequency and smoothing on strain measurements [10–12]. However, there is a lack of studies that assess the effect of drift compensation in neonates. STE is based on tracking acoustic interference patterns over successive imaging frames. However, this tracking is not perfect, and an accumulation of tracking errors over successive imaging frames leads to erroneous measurements of strain, referred to as drift [13, 14]. As a consequence, strain curves do not return to their baseline at the end of the analyzed cardiac cycle. In order to adjust for this effect, speckle tracking software can introduce a correction that returns the strain curve to the baseline at the end of the cardiac cycle. This is called drift compensation, and an example of this is shown in Fig. 1. Clinical guidelines recommend that studies report whether drift compensation is applied or not, and that users should have the option of turning it on or off [15]. However, there is a lack of studies that have quantified the impact of STE drift compensation on strain measurements, or assessed whether the effect of drift compensation depends on the layer of myocardium being assessed or whether the three plane GLS versus 4ch GLS is being applied (an example of three plane GLS is provided in Fig. 2).

The aim of this study is therefore to assess the effect of using the 4ch versus three plane model, different layers and drift compensation on GLS measurements. As each of these parameters may affect each other (for instance, the effect of drift compensation could depend on which layer is being assessed for GLS) we also wish to account for the interplay between these imaging aspects.

## Materials and methods

### Patient population

This was a single center study that prospectively enrolled term born healthy neonates between June 2017 and December 2018 at Haukeland University Hospital, Bergen for a project on perinatal STE. Images were obtained specifically for the project. Routine echocardiography was performed in order to rule out pathology. Informed consent was obtained from the parents in addition to the approval of The Regional Committee for Medical and Health Research Ethics (No. 2015/1918). Inclusion criteria were healthy neonates, healthy being defined as term birth (gestational age 37–42), uncomplicated pregnancy, uncomplicated birth and no other known maternal or fetal/neonatal pathologies. Exclusion criteria were thus any maternal, fetal, or neonatal pathologies as well as preterm or postterm birth. Sample size calculations for paired variables were carried out, α = 0.05 and β = 0.8 [16]. The minimum effect size for strain was set at a difference of 1% GLS, and in order to obtain a reasonable estimate of the expected variability, a pilot study of five neonates were performed in order to gauge the order of magnitude of the expected variability in order to obtain a suitable sample size. A patient dropout buffer of 20% was included.

### Image acquisition and analysis

Twenty five neonates underwent a general echocardiographic examination on the second day post-partum. The neonates were examined in a relaxed supine position using a Vivid E9 scanner with a 12S cardiac probe with transmitted frequency 9 MHz (GE Vingmed Ultrasound, Horten, Norway). For the strain analysis, B mode images of the left ventricle were acquired in the three apical views, namely 4ch, 3ch and 2ch. Sector width and depth were adjusted so that only the complete left ventricle was visible in order to maximize the beam density and frame rate in accordance with the high heart rate of neonates. The images were then analyzed using EchoPac v.202 (GE Vingmed, Horten, Norway). Region of interest for STE was defined by tracing along the endocardial border and defining a thickness that covered the myocardium while excluding the pericardium. The software presents its own assessment of tracking quality. If the software reported faulty tracking in any segment, or if the tracking was otherwise clearly faulty, the image and corresponding patient was excluded. Spatial and temporal smoothing were set at the minimal levels as recommended in previous guidelines [15]. Aortic valve closure was identified through a pulsed Doppler recording. GLS was assessed in all three vendor-defined myocardial layers, namely endocardial, midwall and epicardial. Inter- and intra-observer agreement for GLS strain values was assessed for all the examined patients by having a second observer (T.R.O) analyze all the images blinded to the assessment of the first observer (U.K), followed by the first observer reassessing all the images 1 month later.

### Statistical analysis

For GLS measures, a three way repeated measure ANOVA with Bonferroni post-hoc multiple contrast tests was performed to assess whether GLS was affected by GLS model (4ch or three plane), drift compensation (on or off) and which layer was being assessed (endocardial, midwall or epicardial). Normal distribution was assessed using Shaprio–Wilk test of normality (p > 0.05). The underlying assumption of sphericity was assessed using Mauchly’s test of sphericity, and the Greenhouse–Geisser adjustment was applied when assumption of sphericity was violated (p < 0.05). Agreement was evaluated using two-way mixed interclass correlation coefficients of absolute differences using the classification system proposed by Cicchetti et al. (below 0.40 is “poor”, 0.40–0.59 is “fair”, 0.60–0.74 is “good”, and above 0.75 is “excellent”) [17] and coefficients of variation (root mean square method). Homogeneity of variance was assessed using Bartlett’s test of equal variance. For the segmental strain values, agreement between pairwise measurements of strain when drift compensation was on and off was conducted using Bland–Altman analyses.

## Results

**Fig. 3:**
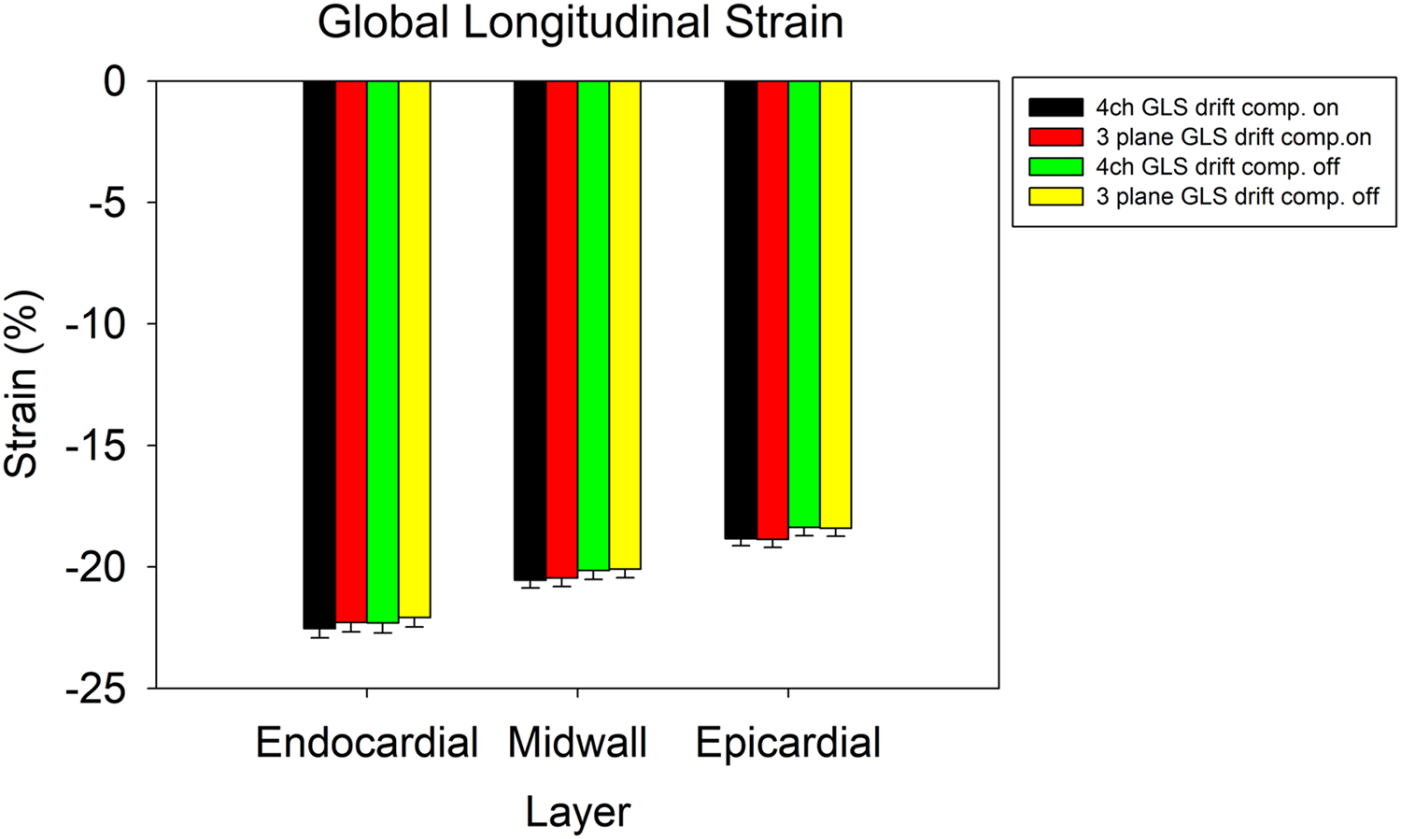
Summary figure displaying global longitudinal strain at different speckle tracking settings. Error bars represent standard error of mean. *GLS* Global longitudinal strain; *4ch* Four chamber

**Fig. 4:**
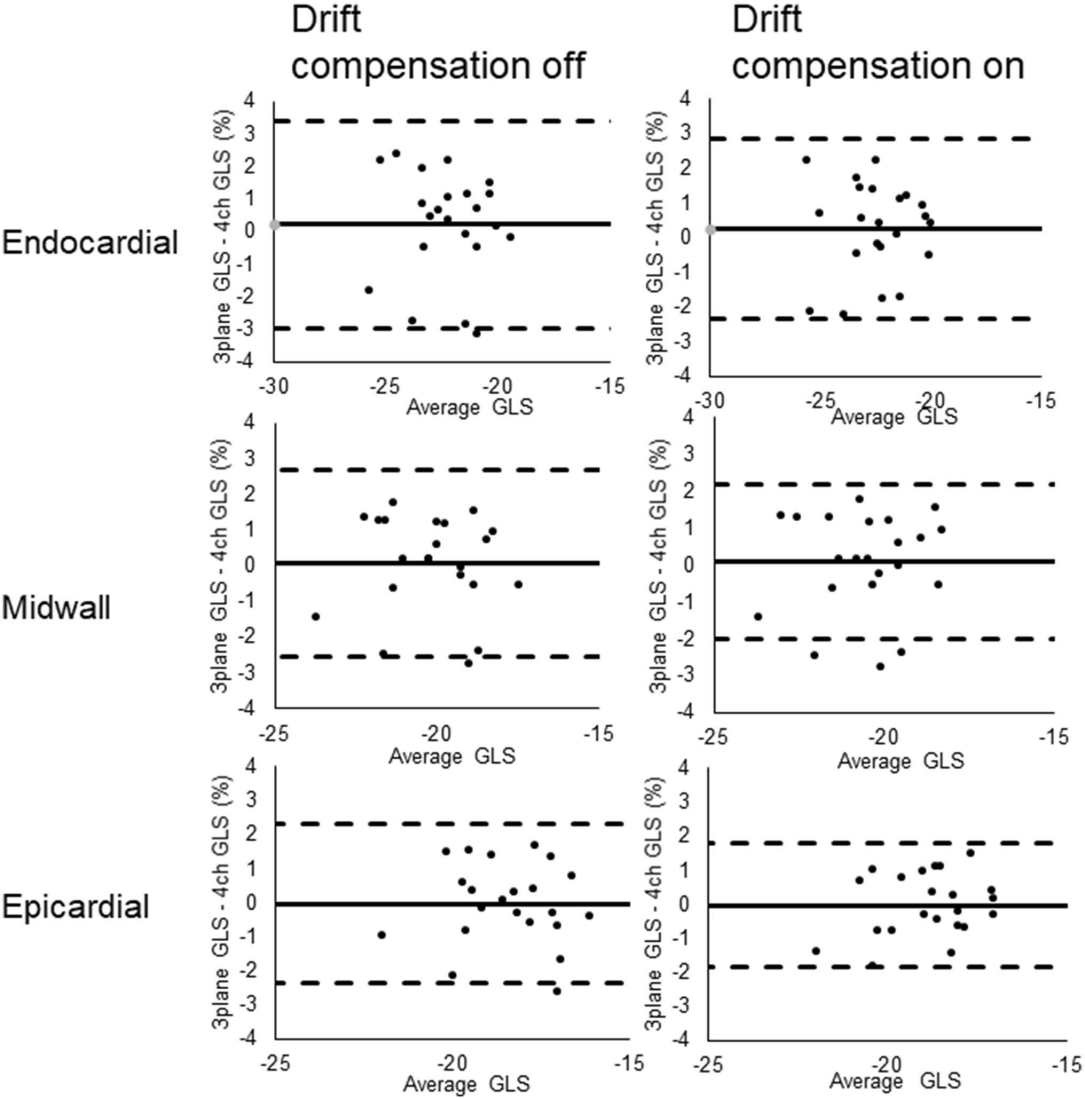
Bland–Altman plots showing agreement between three plane global longitudinal strain versus four chamber global longitudinal strain for different layers and for different settings for drift compensation. Stapled lines represent upper and lower limits of agreement, whereas the continuous line represents mean difference. *GLS* Global longitudinal strain; *4ch* Four chamber

**Fig. 5:**
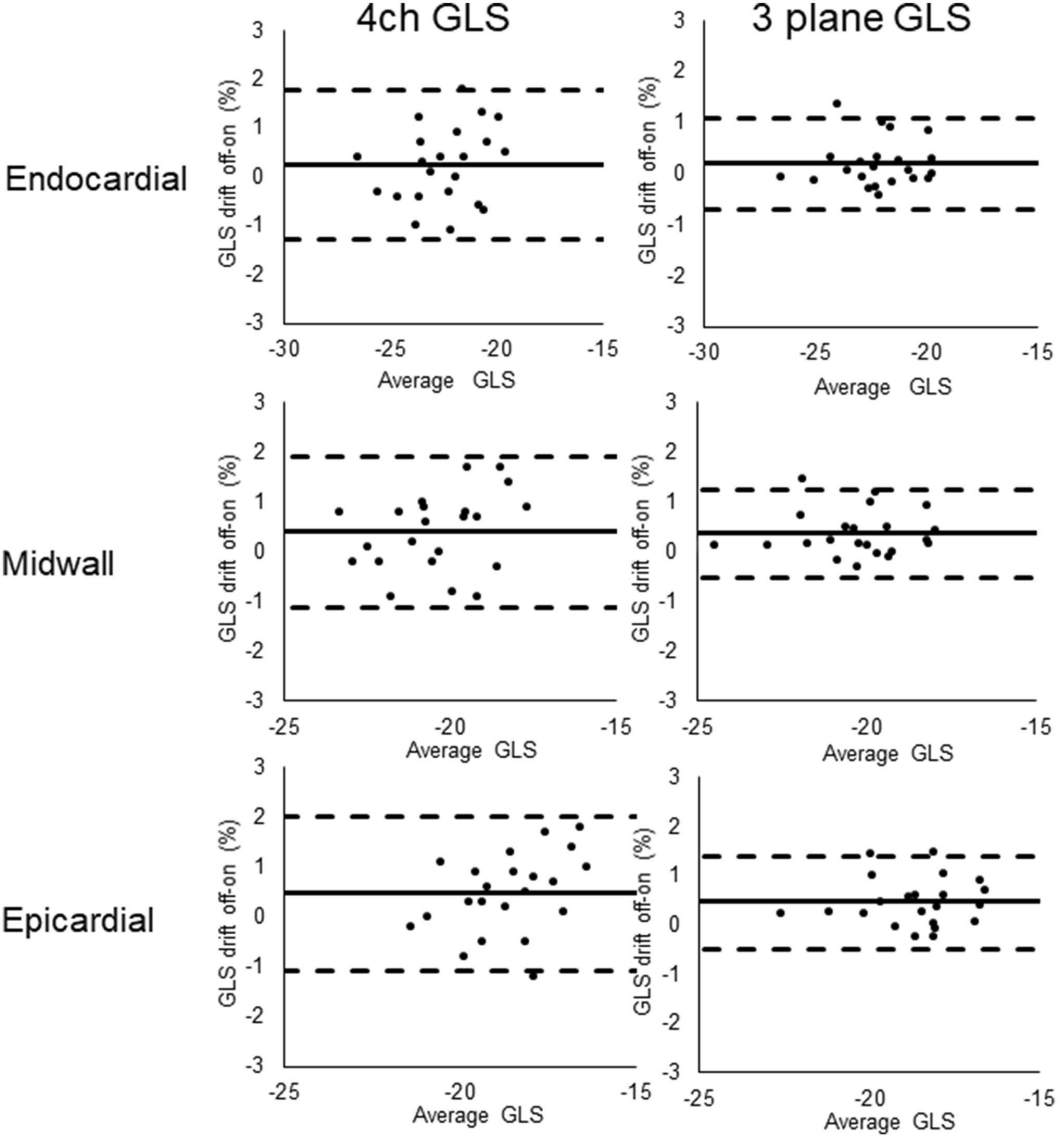
Bland–Altman plots showing agreement between global longitudinal strain when drift compensation off versus on for different layers and for both 4ch global longitudinal strain and three plane global longitudinal strain. Stapled lines represent upper and lower limits of agreement, whereas the continuous line represents mean difference. *GLS* Global longitudinal strain; *4ch* Four chamber

**Fig. 6:**
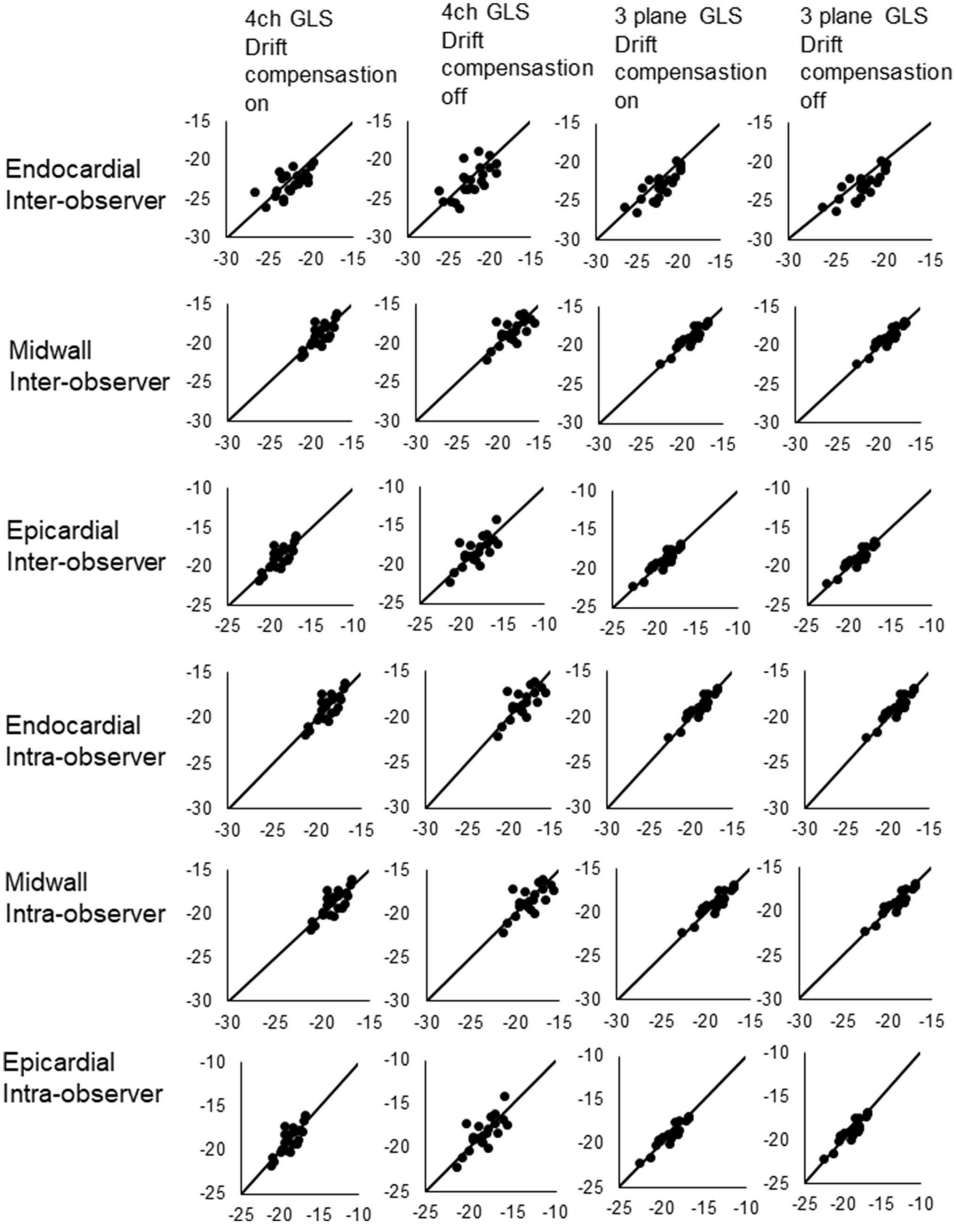
Scatterplots with lines of equality for inter and intraobserver reproducibility of global longitudinal strain for different layers, drift compensation settings and both three plane and four chamber global longitudinal strain. Intraclass correlation values presented in Tables 4 and 5. *GLS* Global longitudinal strain; *4ch* Four chamber

Three patients were excluded on the basis of faulty tracking in the basolateral segment. The characteristics of the remaining neonates are presented in Table 1. Figure 3 presents the values of GLS across the examined settings and models, whereas Table 2 shows the corresponding mean differences and statistical significances derived from the ANOVA. As there was an interaction effect between the effect of drift compensation and wall layer (p = 0.001), Table 3 presents post-hoc analyses for both drift compensation and wall layer. Figures 4 and 5 present corresponding Bland–Altman comparisons of GLS. Bartlett’s test revealed no significant difference in variance (p = 0.9166). Whether one measured 4ch GLS (− 20.5±1.5%) or three plane GLS (− 20.4±1.6%) did not have a statistically significant effect (p = 0.712). This held true irrespective of which wall layer GLS was being measured in. Conversely, endocardial GLS (− 22.3 ± 7.7%) was greater than midwall GLS (− 20.3 ± 7.0%), and midwall GLS was greater than epicardial GLS (− 18.6 ± 6.4%), irrespective of whether a 4ch or a three plane model was used, leading to a gradient in strain of 3.7% from the epicardial to the endocardial layer (depending on whether drift compensation was on or off), p < 0.001. Statistically significant (p = 0.004), albeit small, changes in strain were seen when turning drift compensation on (GLS − 20.6 ± 1.5%) or off (GLS − 20.2 ± 1.6%) depending on which layer was assessed. Figure 6 shows scatterplots for interobserver and intraobserver rerproducibility, with the corresponding Tables 4 and 5 showing intraclass correlation coefficients and coefficient of variation. Inter-observer repeatability was in the good to excellent rage for three plane GLS, whereas it was mostly in the fair to excellent range for the 4ch GLS with the exception of the epicardial GLS which showed a poorer correlation.

**Table 1:** Patient and imaging characteristics

Gender male	50%
Gestational age (weeks ± SD)	40 ± 0.5
Birthweight (grams ± SD)	3650 ± 1075
Heart rate (BPM ± SD)	124 ± 18
Frame rate (FPS ± SD)	167 ± 30
Patent foramen ovale	50%
Patent ductus arteriosus	41%

*BPM* Beats per minute; *FPS* Frames per second; *SD* Standard deviation

**Table 2:** ANOVA of GLS using different image parameters

Image parameter	Mean difference GLS (%)	
Layers		
Endocardial-midwall	− 2.0%	p **< **0.001
Midwall-epicardial	− 1.7%	p **< **0.001
Endocardial–epicardial	− 3.7%	p **< **0.001
Planes		
Three plane GLS-4ch GLS	0.1%	p = 0.712
Drift compens.		
Drift compens. off–on	0.4%	p = 0.004

*ANOVA* Analysis of variance; *Drift compens.* Drift compensation; *GLS* Global longitudinal strain; *4ch* 4 Chamber

**Table 3:** Post-hoc pairwise comparisons ANOVA for drift compensation and wall layer

Image parameter	Mean difference GLS (%)	
Drift compensation on		
Endocardial-midwall	− 1.9%	p < 0.001
Midwall-epicardial	− 1.6%	p < 0.001
Endocardial–epicardial	− 3.6%	p < 0.001
Drift compensation off		
Endocardial-midwall	− 2.1%	p < 0.001
Midwall-epicardial	− 1.7%	p < 0.001
Endocardial–epicardial	− 3.8%	p < 0.001
Endocardial layer		
Drift compens. off–on	0.2%	p = 0.061
Midwall layer		
Drift compens. off–on	0.4%	p = 0.003
Epicardial layer		
Drift compens. off–on	0.5%	p = 0.001

*ANOVA* Analysis of variance; *Drift compens.* Drift compensation; *GLS* Global longitudinal strain

**Table 4:** Reproducibility of three plane and 4ch global longitudinal strain (drift compensation on)

	Layer	ICC (95% CI)	COV (95% CI)
Intra-observer three plane GLS	Endocardial	0.97 (0.92–0.99)	2.0 (1.3–2.5)
	Midwall	0.97 (0.93–0.99)	1.8 (1.3–2.3)
	Epicardial	0.96 (0.91–0.98)	2.1 (1.5–2.6)
Inter-observer three plane GLS	Endocardial	0.86 (0.61–0.95)	3.9 (3.0–4.7)
	Midwall	0.89 (0.73–0.95)	3.4 (2,3–4,2)
	Epicardial	0.85 (0.64–0.93)	3.9 (1.9–5.2)
Intra-observer 4ch GLS	Endocardial	0.91 (0.79–0.96)	3.4 (1.6–4.6)
	Midwall	0.92 (0.80–0.97)	3.1 (1.2–4.3)
	Epicardial	0.89 (0.72–0.95)	3.4 (2.0–4.4)
Inter-observer 4ch GLS	Endocardial	0.78 (0.46–0.90)	4.3 (3.2–5.2)
	Midwall	0.80 (0.53–0.91)	4.1 (2.5–5.3)
	Epicardial	0.67 (0.10–0.87)	5.7 (3.4–7.4)

*COV* Coefficient of variation; *CI* Confidence interval; *GLS* Global longitudinal strain; *ICC* Intraclass correlation coefficient; *4ch* 4 Chamber

**Table 5:** Reproducibility of three plane and 4ch global longitudinal strain (drift compensation off)

	Layer	ICC (95% CI)	COV % (95% CI)
Intra-observer three plane GLS	Endocardial	0.95 (0.89–0.98)	2.4(1.6–3.0)
	Midwall	0.96 (0.89–0.98)	2.4 (1.7–2.9)
	Epicardial	0.94 (0.86–0.98)	2.7 (1.8–3.3)
Inter-observer three plane GLS	Endocardial	0.89 (0.67–0.96)	3.7 (2.4–4.7)
	Midwall	0.90 (0.77–0.96)	3.4 (2.4–4.2)
	Epicardial	0.85 (0.65–0.94)	4.3 (2.7–5.4)
Intra-observer 4ch GLS	Endocardial	0.84 (0.62–0.94)	5.0 (2.6–6.6)
	Midwall	0.86 (0.67–0.94)	4.7 (2.2–6.2)
	Epicardial	0.86 (0.65–0.94)	4.7 (2.7–6.2)
Inter-observer 4ch GLS	Endocardial	0.80 (0.52–0.91)	5.2 (3.5–6.5)
	Midwall	0.79 (0.51–0.91)	5.7 (2.6–7.8)
	Epicardial	0.68 (0.21–0.87)	8.0 (1.6–11.2)

*COV* Coefficient of variation; *CI* Confidence interval; *GLS* Global longitudinal strain; *ICC* Intraclass correlation coefficient; *4ch* 4 Chamber

**Fig. 7:**
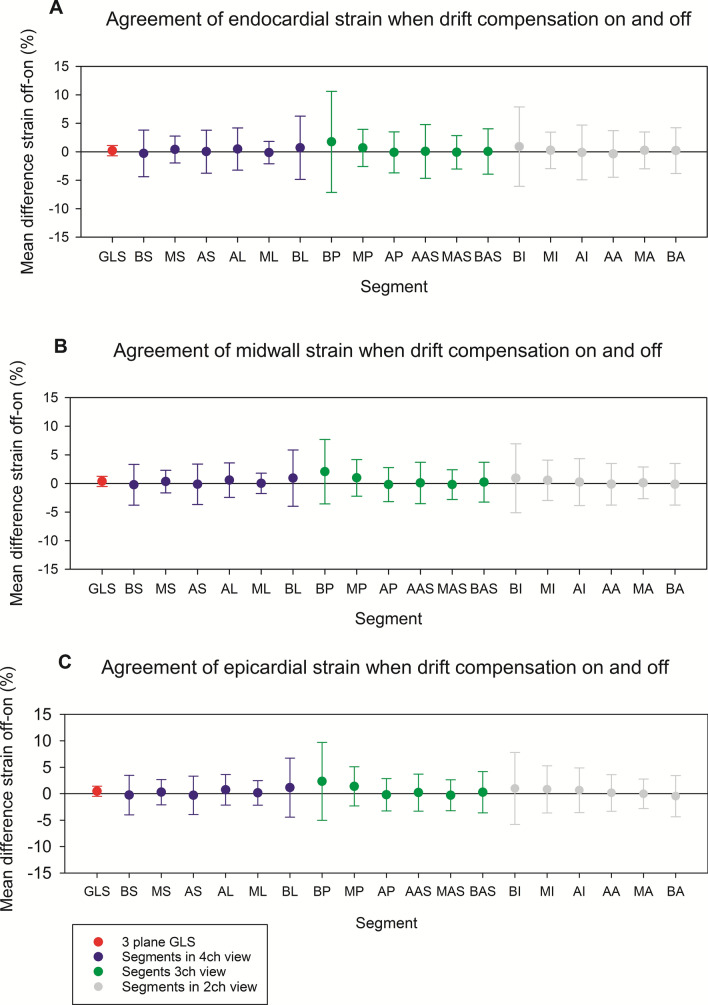
Bland–Altman analyses of pairwise comparisons of strain with drift compensation off versus on in each of the 18 segments. Each point represents the mean difference of a separate Bland–Altman analysis corresponding to a specific segment, with the error bars representing limits of agreement. A: Endocardial layer; B: Midwall; C: Epicardial layer. AA: Apicoanterior; AAS: Anterior apicoseptal; AI: Apicoinferior; AL: Apicolateral; AP: Apicoposterior; AS: Apicoseptal; BA: Basoanterior; BAS: Basoanterior septal; BI: Basalinferior; BL: Basolateral; BP: Basoposterior; BS: Basoseptal; GLS: Global longitudinal strain; MA: Midanterior; MAS: Midanterior septal; MI: Midinferior; ML: Midlateral; MP: Midposterior; MS: Midseptal

In Fig. 7 we see the effect of drift compensation on the segmental strain values as the summary of Bland–Altman plots for each segment. There was greater discrepancy in strain values for when drift compensation was turned on or off for segmental strain values compared to GLS. The greatest discrepancy was seen in the basal segments (basolateral for the 4ch view, basoposterior for the 3ch view and basoinferior for the 2ch view), indicating poorer agreement for these segments.

## Discussion

The primary findings of this study are that GLS values in healthy neonates are robust with regards to whether 4ch GLS or three plane GLS is assessed and whether drift compensation is on or off. This in turn indicates that reference values of GLS are unlikely to be substantially affected by these factors.

Although guidelines recommend the use of three plane GLS, 4ch GLS is often used instead [6, 8, 15]. In recent years, studies on adults have begun to emerge that compare three plane GLS against 4ch GLS. One such study showed good agreement and similar prognostic value of 4ch GLS and three plane GLS in heart failure patients [18]. Another study aimed at detecting cardiotoxicity was conducted in patients undergoing chemotherapy [19]. This study also found good agreement between three plane GLS and 4ch GLS, but nuanced this by showing some discordance regarding threshold values of cardiotoxicity for these two models in addition to lower reproducibility for 4ch GLS.

There is a shortage of studies in healthy pediatric cohorts that compare 4ch and three plane GLS. However, previous meta-analyses of normal GLS values in healthy children have included studies that present both 4ch and three plane GLS. Whereas some meta-analyses across the pediatric age range did not find any difference between 4ch and three plane GLS [8, 20], a smaller meta-analysis of neonates found that three plane GLS tended to be higher than 4ch GLS [6]. This neonatal meta-analysis, however, only had five studies in each sub-group. Furthermore, the included studies were carried out by different clinicians using different equipment and acquisition settings, which could confound the results.

This study indicates that 4ch GLS and three plane GLS do not differ in healthy neonates. The natural follow up question is then whether 4ch GLS and three plane GLS can be used interchangeably in a clinical setting. This would be of great interest, as obtaining 4ch GLS is faster than obtaining three plane GLS. This is particularly true in the context of neonatal cardiology as the neonates are frequently restless and sometimes critically ill. Previous studies have shown the clinical utility of both three plane [21–23] and 4ch GLS [7, 24, 25] in neonates. Neonatal and pediatric cardiology involves assessment of a range of complex structural congenital heart diseases. Currently, there is a lack of studies that compare 4ch GLS and three plane GLS in such patients. However, we know that the 4ch view assesses a very limited portion of the left ventricle, whereas three plane GLS assessed a larger, though still limited, portion. For heart conditions that evenly affect ventricular function, 4ch GLS is possibly a reasonable measure of GLS. However, if there is an asymmetric effect on the ventricle, there is a greater probability for 4ch GLS and three plane GLS diverging. Ultimately, however, agreement between 4ch and three plane GLS as well as their correlation with clinical endpoints would have to be assessed for the pathology in question in order to compare their clinical interchangeability.

Although confidence intervals overlapped, reproducibility of three plane GLS tended towards higher values than 4ch GLS. One likely explanation is that three plane GLS is averaged over three cardiac cycles as three different views are being assessed. A previous study in neonates has shown that averaging 4ch strain across three cardiac cycles improved reproducibility of longitudinal strain measurements [20]. Hence, a potential alternative to three plane GLS could be obtaining 4ch GLS over several successive cardiac cycles and averaging strain across these cycles.

This is the first study to have quantitatively assessed the effect of drift compensation on STE derived strain measurements in neonates. In order to account for variability in strain measurements, there has been an ongoing effort to standardize measurements across vendors and identify which factors introduce variability in strain measurements. This is necessary for optimal acquisition of strain across age groups. This effort can, in broad terms, be divided into studies that deal with image acquisition variables such as frame rate [10] and frequency [26] on one hand, and studies dealing with image processing variables such as smoothing [12] and vendor heterogeneity in the speckle tracking software on the other hand [11]. Previous studies in adults have indicated that it is image processing rather than image acquisition that is the primary source of variability [27]. Therefore, much effort is put into examining inter-vendor software differences as well as the utility of vendor-independent software [28]. However, there is a lack of studies into user-regulated software settings such as turning drift compensation on or off. This study aimed to address this knowledge gap. It is especially important to conduct such studies on neonates, as they have proven to be a particularly difficult group with regards to speckle tracking, and constitute an age group where normal values in healthy subjects is still being established [6, 29]. We found that drift compensation had a negligible effect on the mean values of both three plane and 4ch GLS.

Although assessment of segmental strain values was not a central aspect of this paper, it is interesting to note that the segmental strain values displayed larger dispersion when drift compensation was turned on or off compared to three plane GLS. This is likely due to the fact that on a segmental level, turning drift compensation on or off can both increase and decrease strain. By averaging strain across segments, these changes even out, resulting in a smaller degree of dispersion. The segments that displayed the greatest dispersion were the basolateral segment in the 4ch view, basoposterior in the 3ch view and basoinferior in the 2ch view. This could be due to decreased lateral spatial resolution at increased distances from the probe as well as noise resulting from reflections and scattering in the pericardium. Previous studies have shown greater variability and higher susceptibility to smoothing settings for regional strain measurements compared to GLS [12, 30]. This study adds to this knowledge by indicating that segmental strain values are more sensitive to drift compensation settings than GLS.

Although speckle tracking derived multilayer strain has been available for a decade, it has received increased attention in recent years, and studies have also emerged that show its clinical applicability and utility in children [31–33]. However, there is a shortage of studies that examine multilayer strain in neonates. Our study shows increasing GLS when moving from the epicardial towards the endocardial layer as well as reduced reproducibility of GLS in the epicardial layer compared to the other layers, corresponding well to adult studies [9].

This study examined longitudinal strain as this is the most commonly assessed strain direction [6]. GLS is also recommended as a measure of global left ventricular function [2]. The other two strain directions, circumferential and radial strain, are derived from the short axis views rather than the long axis views. Longitudinal strain is both easier to obtain and more reproducible than the other strain directions, which contribute to its role as the strain direction of choice [34]. Nonetheless, the other two strain directions are gaining traction, and it is possible that they will gradually play a larger role in echocardiographic assessments of ventricular function [35]. Previous studies have shown that image acquisition settings have a different effect on strain obtained in the short axis views compared to strain obtained in the long axis views [26]. Therefore, future studies assessing the effect of image acquisition and processing parameters on short axis view derived strain measurements are warranted.

A repeated measure ANOVA was used in order to account for hemodynamic loading conditions. As the same ultrasound images were assessed using different settings we were able to account for hemodynamic conditions such as load and heart rate.

Our study only assessed longitudinal systolic strain and only used EchoPac software as this is the most commonly assessed strain parameter and most commonly used speckle tracking software, respectively [6]. This study also lacked a reference method such as sonomicrometers or MRI against which one could assess the accuracy of STE values. Such assessments are difficult in neonates. This study only assessed a small cohort of healthy neonates, and thus should be interpreted with caution with regards to older subjects or in case of cardiac pathology.

## Conclusion

This study indicates that STE GLS in healthy neonates is robust both with regards to the use of 4ch GLS versus three plane GLS as well as the use of drift compensation. GLS increases as one moves from the epicardial layer towards the endocardial layer. For segmental strain values, drift compensation introduces significant variability in the data.

## Data Availability

Data available on reasonable request from authors.
